# The Application of Chitosan Nanostructures in Stomatology

**DOI:** 10.3390/molecules26206315

**Published:** 2021-10-19

**Authors:** Shunli Chu, Jue Wang, Fengxiang Gao

**Affiliations:** 1 Department of Implantology, Hospital of Stomatology, Jilin University, Changchun 130000, China; chusl@jlu.edu.cn; 2 Department of Prosthodontics, Hospital of Stomatology, Jilin University, Changchun 130000, China; wangjue19@mails.jlu.edu.cn; 3 Changchun Institute of Applied Chemistry, Chinese Academy of Sciences, Changchun 130000, China

**Keywords:** nano-chitosan, tissue engineering, drug carry, remineralization, root canal treatment

## Abstract

Chitosan (CS) is a natural polymer with a positive charge, a deacetylated derivative of chitin. Chitosan nanostructures (nano-CS) have received increasing interest due to their potential applications and remarkable properties. They offer advantages in stomatology due to their excellent biocompatibility, their antibacterial properties, and their biodegradability. Nano-CSs can be applied as drug carriers for soft tissue diseases, bone tissue engineering and dental hard tissue remineralization; furthermore, they have been used in endodontics due to their antibacterial properties; and, finally, nano-CS can improve the adhesion and mechanical properties of dental-restorative materials due to their physical blend and chemical combinations. In this review, recent developments in the application of nano-CS for stomatology are summarized, with an emphasis on nano-CS’s performance characteristics in different application fields. Moreover, the challenges posed by and the future trends in its application are assessed.

## 1. Introduction

The oral cavity is a complex microenvironment that is vulnerable to various physical, chemical, and microbial injuries, resulting in oral diseases. These include soft tissue diseases, such as gingivitis, aphthous ulcers, and other mucosal diseases; hard tissue diseases, such as caries, fractures, and bone defects; and combined soft and hard tissue diseases, such as periodontitis and tumors. Therefore, in stomatology, it is necessary to find a medical material that features different characteristics under different treatment conditions. In recent years, many researchers have found that nano-chitosan offers application advantages against various oral diseases [1,2,3,4,5,6].

**Figure 1:**
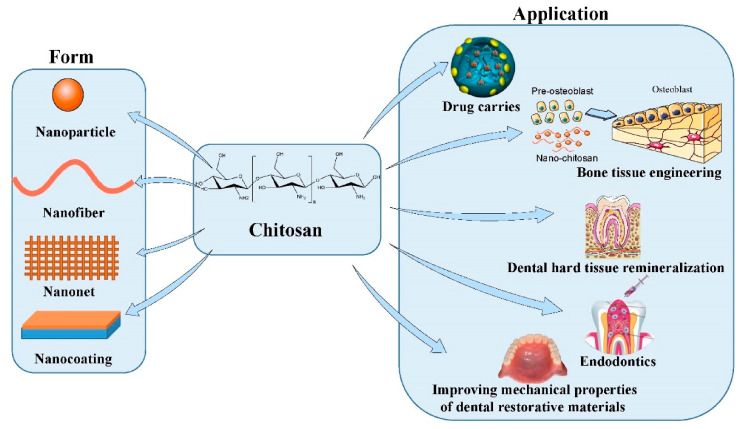
Common forms of nano-chitosan and their application in stomatology (Picture material cited from https://smart.servier.com/ (accessed on 10 September 2021) and [4]).

Chitosan (CS) is a cationic polymer composed of β-(1-4)-linked d-glucosamine and *N*-acetyl-d-glucosamine [7,8]. The cationic properties of CS enable it to combine with polyanions to form complexes; it also features gelation characteristics [9]. Furthermore, many of the characteristics of CS, such as its low water and acid solubility, good biodegradability, good biocompatibility, non-toxicity, antibacterial ability, anti-plaque effects, and anti-adhesion properties, allow the application of CS in many fields, especially in stomatology [7,9,10]. Intermolecular hydrogen bonding enables nano-CS to form stable nanogels, which feature a smaller size and higher specific surface area than CS [10,11,12,13]. Moreover, nanoscale confers certain characteristics upon nano-CS that are not present in CS. These characteristics include higher permeability, better biocompatibility, higher charge density, and greater support for the development of cells. These unique characteristics of nano-CS enable its abundant applications in stomatology (shown in Figure 1). This review focuses on the preparation of CS nanoparticles and the antibacterial properties of nano-CS. We also consider the application of nano-CS in improving the mechanical properties of dental-restorative materials, bone tissue engineering, dental hard tissue engineering, targeted drug carrying for soft tissue and root canal treatment, and in the mechanical properties of dental-restorative materials.

## 2. Application of Nano-CS as Drug Carriers in Oral Soft Tissue Diseases

In different treatment methods for oral diseases (such as mucosal diseases, periodontitis, etc.), local drug administration with targeted- and sustained-release characteristics is preferable to systemic administration. Local administration can reduce the toxicity and side effects of drugs that are absorbed by other tissues or organs [4,14]. However, determining how locally administered drugs can be retained in the targeted tissue for a long time and released slowly in the presence of saliva and food-chewing remains challenging [4].

Chitosan is often used as a carrier for targeted drug delivery, to sustain drug effects at a subcellular scale, to achieve cellular targets with high accuracy, to achieve maximum therapeutic effect, and to decrease adverse effects [15,16,17]. In this approach, active drug substances are dissolved, entrapped, or encapsulated and absorbed or attached to the drug carriers [16,18]. Cationic CS can be electrostatically adsorbed with mucin carboxyl groups on the mucosal and enamel surface, in order to stay in the oral cavity for more than 6 h, and the film-forming ability of CS makes the drugs or biomolecules it is carrying release slowly [3,4,18,19]. When the pH of the oral environment is lower than 6.5 (the ionization constant of CS), CS can dissolve in water and release any drug or bioactive molecule it is carrying [20]. For instance, nano-CS has the ability to stably and continuously increase the release of NaF in an acidic environment with a pH of 5 to 7 [1,20]. Therefore, as a drug carrier, CS has considerable application prospects in acidic oral microenvironments. The small size of nanoscale increases the probability of biological membrane penetration by drugs carried by nano drug carriers, significantly increasing the bioavailability of the drugs and reducing their toxicity and side effects [17,21]. For example, compared to silver diamine fluoride alone, nano silver fluoride (NSF), which is composed of nanoparticles of silver and CS, has a lower effective dose against *Streptococcus mutans* and lower toxicity [22].

Nano-CS may have many effects in local oral administration, such as targeted adhesion to the surface of oral tissues, slow drug release, resistance to acidic oral environments, the improvement of drug bioavailability, and so on.

**Figure 2:**
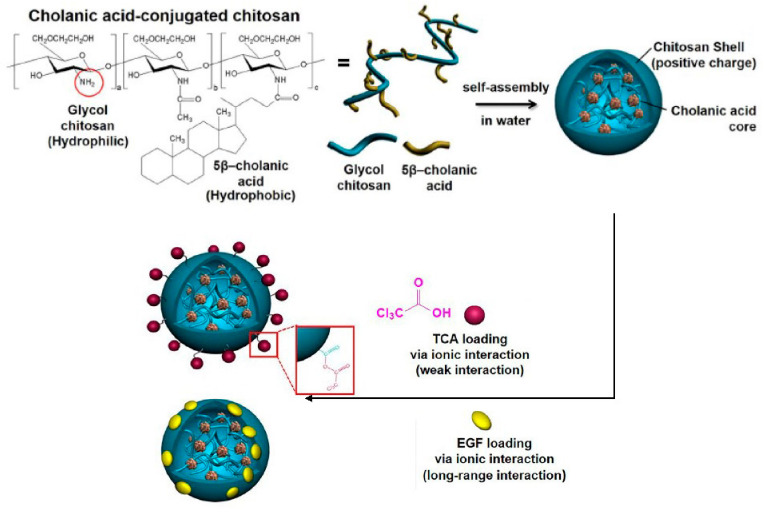
Preparation of HGC-based nano-controlled release system. CS was conjugated with hydrophobic cholanic acid. Hydrophobic cholanic acid is located inside the nanoparticles in water, and the hydrophilic cationic polymer amine group (NH_2_) of CS forms the structure of the nanoparticles. Cationic CS loads anions, TCA, and EGF through ionic bonds. Reprinted with permission from ref. [17]. Copyright 2021 Springer Nature.

**Figure 3:**
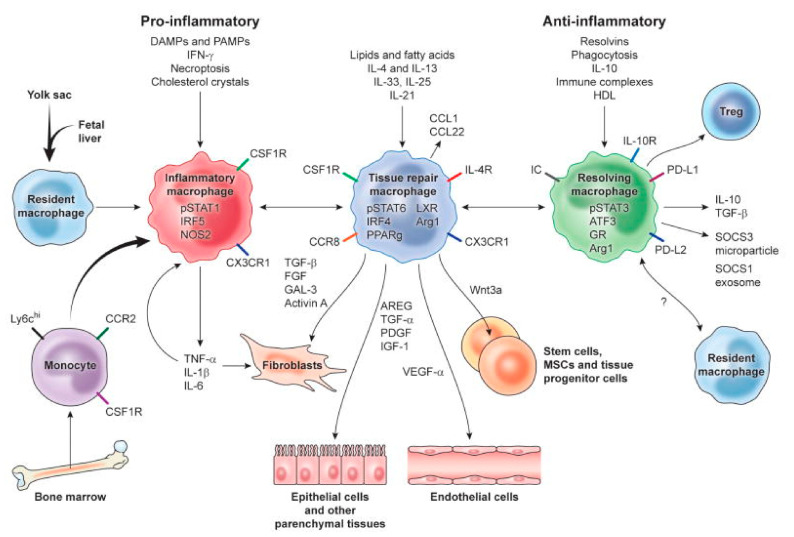
Mechanisms of major macrophage activation phenotypes in tissue repair, regeneration, and fibrosis. Tissue-resident and recruited macrophages have phenotypic and functional changes when biological factors are released in the local tissue microenvironment. M2 phenotype macrophages (green) can produce various biological processes to stimulate cell proliferation, differentiation, and activation, in order to promote tissue repair. Reprinted with permission from ref. [40]. Copyright 2021 Elsevier.

Nano-CS obtained by gamma irradiation, which is covered by bacterial cellulose, could significantly inhibit microbial strains in difficult-to-heal oral wounds [23]. This antimicrobial activity can inhibit bacterial invasion to protect the wound from secondary infection [23]. Nano-CS has a disc-like shape and an average diameter in the range of 40 to 60 nm. However, the release of nano-CS from BC is slow and continuous [23]. Tee [11] formed nano-CS loaded with recombinant human keratinocyte growth factor (nano-CS/rHuKGF) to increase the stability of rHuKGF and prevent rHuKGF proteolysis in saliva. Hydroxy-modified glucose CS (HGC) nanoparticles were synthesized by conjugating hydrophilic glycol CS with hydrophobic β-cholanic acid, and the synthesized nanoparticles showed good solubility in neutral solution, could self-assemble in neutral solution, and could encapsulate drugs for sustained release [24,25]. The core of the HGC nanoparticles was composed of hydrophobic β-cholanic acid covered by a hydrophilic cationic CS shell (shown in Figure 2). Drugs such as anionic trichloroacetic acid (TCA) and epidermal growth factor (EGF) can be loaded through ionic bonds (shown in Figure 3) [17,26]. The nano-controlled release system significantly reduced the danger of applying TCA locally [17]. Further, the nano-controlled release system upregulated the cell survival genes of the PI3K-AKT signaling pathway [26]. The system significantly increased the expression of gingival growth factors and soft tissue growth-related genes and significantly promoted soft tissue regeneration in animal experiments [17]. Therefore, HGC nanoparticles are an ideal nano-controlled release system for improving soft tissue regeneration. Doxycycline covered by nano-CS was utilized as an adjunct to basic periodontal therapy for moderate chronic periodontitis. Compared to traditional doxycycline and placebo-CS, gingival crevicular fluid levels of interleukin (IL)-6 and tumor necrosing factor-α significantly decreased, which was thought to have resulted from reduced inflammatory processes associated with tissue destruction in periodontal pockets. As a local delivery system, doxycycline covered by nano-CS is easy to apply and insert, which is suitable for the dimensions of the pocket, and it is easy to access the bottom of the pocket, reducing the pain of ingesting the medicine [27].

## 3. Application of Nano-CS in Bone Tissue Engineering

The presence of tumors, trauma, and other diseases can lead to oral and maxillofacial bone tissue defects. A large maxillofacial bone tissue defect not only affects patients’ chewing, speech, and other functions, it also has a significant negative impact on their appearance and psychology. Finding bone defect repair materials with good mechanical strength, similar to that of a bone, that can adapt to the shape of the bone defect and promote bone tissue regeneration, is an urgent problem in stomatology. With the in-depth study of bone repair processes and progress in the preparation and characterization of bone repair materials, various artificial bone substitutes have been integrated in the treatment of bone-related diseases [28,29].

Bone tissue engineering deals involves the use of bone substitutes. Scaffolds, stem cells, and bioactive factors are important components of tissue engineering [30,31,32,33,34,35,36]. A favorable surface of scaffolds is required for stem cell attachment, which is the first step in cellular activities, affecting cellular functions [30]. Positively charged CS can promote osteogenesis and angiogenesis activities in adjacent cells [30]. Furthermore, CS is biocompatible and biodegradable. Notably, the active group of CS can combine with inorganic ions in body fluid to serve as a nucleation site for the formation of hydroxyapatite (HAP) (the main inorganic component of hard tissue) [37]. At the same time, CS was found to promote the transformation of macrophages into M2 phenotype to promote anti-inflammatory response and upregulate the secretion of cytokines to activate tissue repair (shown in Figure 3) [38,39,40]. All kinds of cells recruited to the defect site can secrete BMP, PDGF, TGF-β, and other substances to promote healing and trigger a series of cellular reactions, such as the proliferation and differentiation of mesenchymal stem cells and the proliferation of osteoblasts, to promote bone tissue regeneration, in order to organize the hematoma into fibrous tissue [41]. Therefore, CS is an ideal choice for bone tissue engineering scaffolds, in terms of the initiation of cell recognition, the promotion of cellular adhesion and the formation of M2 phenotype macrophages [30,31,32,33,34,36,42,43,44]. However, there are some drawbacks to the use of CS as bone regeneration scaffolds [34,43,45,46].

The mechanical strength of CS alone as a bone tissue scaffold is not sufficient to meet clinical needs. To overcome this drawback, researchers have focused on preparing CS in nanoform and mixing CS with HAP (HAP has a synergetic effect when combined with CS in terms of osteo-inductivity) [2,31,32,33,34,35,43,47,48,49,50,51,52,53,54]. Due to the presence of nano-sized HAP and nano fibrous collagens in the extracellular matrix of hard tissue, scaffolds with nanoscale structures favor tissue engineering [37,55]. Nanoscale CS has a larger surface area and an irregular surface, which can promote cellular adhesion, proliferation, and diffusion, providing favorable conditions for subsequent cell activities [56,57]. HAP/CS nanocomposites can be obtained using in situ hybridization [50,51,58]. Using this method, Ca(NO_3_)_2_ solution and (NH_4_)_2_HPO_4_ solution were successively added to CS solution to obtain a homogeneous polymer solution. Subsequently, the pH was adjusted to approximately 11, and the suspension was centrifuged to obtain a white gel-like precipitate. Finally, nanopowders were obtained by freeze-drying [51,58]. Elkholy found that β-CS/HAP had the ability to promote bone generation and accelerate the formation of Haversian systems in vivo. Compared to control samples, the group with a β-CS/HAP ratio of 30/70 had thicker regenerated trabecular bone, a higher degree of organization, and higher maturity [50]. The silanol group (as the nucleation site) and calcium salt (as the HAP promoter) were introduced in CS/HAP to form a bioactive CS nano-hybrid material [59].

In addition to HAP, researchers have also combined nano-CS with other polymers to further improve the application prospects of CS in bone tissue engineering. For instance, the co-polymerization of gelatin, which features an integrin-binding RGD-like sequence, and CS can increase the compressive properties and cellular attachment of the material [42,60]. The combined scaffold can be produced in a solid shape to adapt to different shapes of bone defects [50]. A 3D tripolymeric scaffold of nano-CS/SF/hyaluronic acid (HA) has higher application potential for tissue engineering than nano-CS alone [55]. Dropwise negatively charged TPP solution was added to an homogeneous CS solution and stirred to obtain nano-CS. The ionic bond formed between TPP and CS strengthens the nano-CS matrix [55,61]. CS can interact with SF through hydrogen bonds to promote the transformation of SF conformation from a random coil to a β-sheet structure, forming a film, which is important in tissue engineering [62]. The combination of the polymers formed a network with an average pore size of 0.5–1 μm, which was suitable for cellular adherence and growth. The scaffold had a highly amorphous structure and a highly functional surface area, which is conducive to the early adhesion, growth, proliferation, and osteogenic differentiation of pre-osteoblast MC3T3-E1 cells [55].

As previously described, bioactive molecules are important. Introducing these molecules into tissue engineering is one of the strategies for establishing physical or chemical bonds between appropriate polymer functional groups and bioactive molecules [50,63]. Shrestha designed a core-shell nano-system that can release transforming growth factor-β1 (TGF-β1) and dexamethasone (Dex) in a time-controlled manner. TGF-β1 was carried by alginate in the core of nanoparticles and Dex was carried by CS in the outer layer. In in vivo experiments, the TD-NS group had a significantly higher (*p* < 0.05) osteo/odontogenic differentiation than the control and free TGF-β1 groups [63].

Natural bone, cementum, and dentin are composed of organic fibers and inorganic apatite, which are dispersed among the fibers [64,65]. Besides the large surface area, which is advantageous for cellular adhesion, interconnected pores in synthetic nanofibers can be a path for nutrients and the metabolic waste of cells [37,47]. Therefore, some researchers have begun to develop CS into nanofibers in recent years [37,47,64]. CS nanofibers, with an average diameter of 140 nm, loaded with sphere-like β-tricalcium phosphate crystallites of an average diameter of 350 nm, are prominent biodegradable scaffolds. In one study, a hybrid nanofiber had a higher osteo-conductivity than a CS nanofiber alone [37]. Calcium silicate/CS complex was dropped on the poly (lactic acid) mat surface to coat nanofibers. The coated nanofibers were confirmed to have the ability to promote cell adhesion and proliferation, which led to better osteogenesis [64]. Nano-HAP and nano silica-incorporated poly(ɛ-caprolactone)-poly (ethylene glycol)-poly (ɛ-caprolactone) (PCEC)-CS nanofibers had a higher tensile strength than CS nanofibers. Meanwhile, nanofibers with nano-HAP were superior to nano silica. The hydrophilicity and the ability of nano silicon-incorporated PCEC-CS nanofibers to promote cellular adhesion and proliferation weas significantly higher than that of nano-HAP-incorporated nanofibers. However, both nano-HAP and nano silicon-incorporated PCEC-CS had osteo/odonto-inductivity to human dental pulp stem cells. Hokmabad concluded that 15 wt% nano-HAP-incorporated PCEC-CS nanofibers had a better ability to promote cell adhesion and differentiation and are potential scaffolds for bone tissue engineering [47].

**Figure 4:**
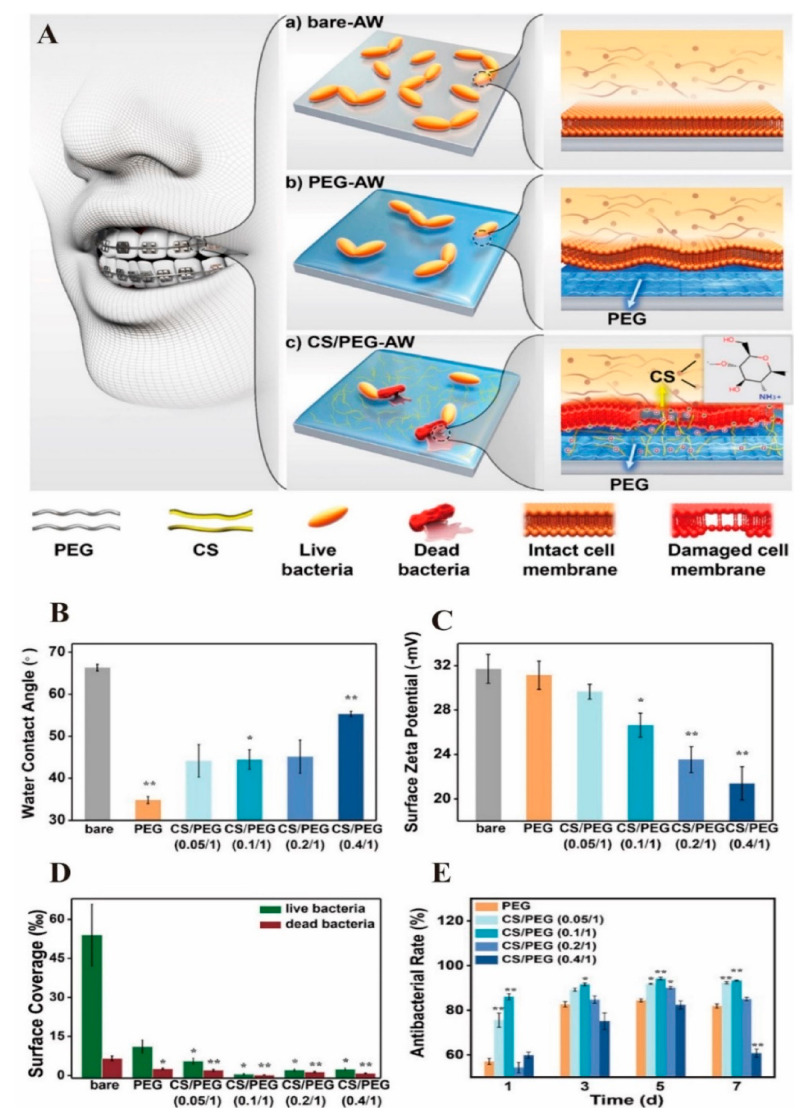
(**A**) Schematic diagram of antiadhesive and antibacterial composite hydrogel coatings: (**a**) Bacteria accumulated significantly on bare stainless steel AW, (**b**) PEGylation of stainless steel AW can significantly reduce bacterial adhesion, (**c**) stainless steel AW with CS/PEG hydrogel coating has the ability to inhibit adhesion and antibacterial activity; (**B**) AW surface contact angle change; (**C**) Surface zeta potential change; (**D**) Surface coverage of live and dead bacteria on materials; (**E**) Rate of long-term antibacterial effect against *S. mutans*. Reprinted with permission from ref. [69]. Copyright 2021 American Chemical Society. * *p* < 0.05; ** *p* < 0.01 from data obtained in the bare-AW group.

Nano-CS can also be used to coat the surface of oral implants to improve their surface properties and to enable the implant surfaces form ideal bone combinations with the surrounding alveolar bone [66]. Nano-CS does not exhibit cytotoxicity and is hydrophilic when it is used as a coating material, allowing proteins related to cell attachment, such as fibronectin, to attach and spread [66,67]. Meanwhile, nano-CS coating could inhibit the corrosion of Ni-Cr dental alloys, which is caused by *S. mutans* and may produce toxic products or affect patients who are susceptible to allergies [68]. Therefore, coating with nano-CSs expands the application of metals in the field of medicine. To promote corrosion resistance, a nanocomposite was prepared by blending CS/HA/TiO_2_. The impermeable coating formed by this nanocomposite can isolate the alloy and artificial saliva, preventing electron and ion transfer between them [68]. A nanoscale network cross-linked gel structure on the stainless steel archwire and the AW surface, prepared through the alkylation and subsequent polymerization reaction of PEG and CS, had antibacterial properties. When the PEG/CS mass ratio was 1/0.1, combined with the influence of surface material wettability and surface point position, the nano PEG/CS coating exhibited the best antibacterial performance and showed significant long-term colony inhibition efficiency (93.3%, 7 days) (Figure 4) [69]. The CaP/CS coating was prepared by incubating the spin-coated CS layer on the titanium substrate in modified simulated body fluid after 1M NaOH post-treatment, and the hydrophilicity of the sample was significantly increased and the apatite morphology was also transferred onto ellipsoidal clusters [70]. Compared to the HA/CS hybrid coating, the CaP/CS hybrid coating demonstrated better cell viability, adhesion, and differentiation of MG-63 cells [70]. This is consistent with the belief that ACP is more suitable for bone engineering than HAP in vivo, in terms of its better bioactivity, high cell adhesion, adjustable biodegradation, and osteo-conductivity [71].

There are many methods for obtaining nano-CS coating, including passive and electrostatic methods [72]. The passive methods comprise freeze drying, solution casting, and spin coating [70,72], which feature unavoidable disadvantages, such as the inability to uniformly coat complex surfaces and difficulty in controlling the coating thickness, which can be overcome by electrostatic methods [66,70,72,73,74,75,76,77,78]. Notably, 770−800 nm thick nano-HAP/CS coatings without cracks were produced on two-phase (α + β) Ti6Al7Nb titanium alloy substrates by electrophoretic deposition [73,78,79,80]. Electrospray technology ensures irregular implant surfaces, with uniform coating, in the form of nanoparticles [73,81,82,83,84]. The shapes and structures of continuous ultrafine fibers, obtained through electrospinning, can be controlled [47,54,85,86]. To synthesize nanofibers, the prepared nano-CS can be mixed with a combination of other materials, such as poly(vinyl alcohol) (which improves the tenacity and fiber-forming ability), montmorillonite clay (which increases the pore formed during electrospinning), silicon dioxide (which induces the osteo-inductivity of the fibers), and nano-HAP (which increases the surface roughness) [7,47,54]. Nanofiber made of CS with embedded HAP and graphene nanosheets (GNS) through electrospinning on the surface of GNS and Si_3_N_4_ (SN) binary powder-reinforced hybrid titanium metal composite surfaces formed a uniform structure. Moreover, hybrid nanofiber coatings offered superior antibacterial properties against E.coli to other mixtures [54].

However, research on nano-CS as an implant surface coating has mostly focused on the coating technology and its antibacterial properties. Whether nano-CS can promote the formation of good combinations between dental implants and the alveolar bone remains to be explored. However, nano-CS coating technology with antibacterial properties can be applied in orthodontics, metal crowns, and other metal surface treatments for the oral cavity to reduce plaque accumulation and prevent caries, periodontitis, and other oral diseases.

## 4. Application of Nano-CS in Dental Hard Tissue Remineralization

Net mineral loss from a broken balance between demineralization and remineralization causes dental caries, which is an infectious multifactorial disease [87,88]. Conventional strategies for dental hard tissue restoration include the mechanical removal of decay and filling with artificial materials [89]. However, conventional strategies feature drawbacks, such as microleakage and the development of secondary caries caused by microgaps and residual bacteria [87]. In addition to caries, etching and enamel hypoplasia are common [90,91,92]. Traditional treatments for these diseases are not suitable for large-scale erosion [92]. Therefore, drugs or materials that are used to prevent, treat, or repair dental caries should have the triple effect of inhibiting the demineralization of residual dental hard tissue, promoting mineral deposition, and inhibiting cariogenic bacteria. Nano-CS has become a candidate material because of its antibacterial properties and its ability to chelate mineral ions [87]. Mouthwash solutions containing nanoparticles of CS and calcium significantly reduced the oral bacterial colonies in primary school students. Moreover, the number of colonies was inversely proportional to the mouthwash concentration [93]. Nano-CS containing fluoride ions is added to mouthwash water, so that fluoride ions can be slowly released onto the surface of demineralized tooth tissue for extended periods, to promote remineralization [94]. Small-sized nano-CS (1−100 nm) is highly reactive, with better ionic exchange than that of large-sized nano-CS, to form fluorapatite with better acid-resisting ability [18,95].

### 4.1. Enamel Remineralization

In mature enamel, single slender nano-HAP crystals are arranged in parallel, forming a diameter of 3−5 μm and extending perpendicular to the enamel surface. Rods and interrods are intertwined to form a “fish scale-like” structure at an angle of 60° [96,97]. This ordered-oriented mineralization pattern is highly related to the mechanical properties of the enamel. Therefore, it is very important to repair the enamel and retain its mechanical properties [98,99]. Amelogenin (AMEL), non-amelogenin, and other proteins can stabilize ACP and reduce the supersaturation of the remineralization solution, thus inhibiting the growth of crystals, to allow sufficient time for mineralized ions to enter the deep layer of lesions and finally transform into oriented, slender, organized HAP crystals [90,100,101]. AMEL is difficult to obtain and expensive; thus, it is very important to find AMEL alternatives.

Positively charged CS can adhere to negatively charged enamel surfaces. The amino group of CS has a high reactivity to acid; thus, CS can maintain the pH of the oral cavity by maintaining the pH of the plaque above the critical pH and protecting the HAP of the enamel from dissolution [15]. Mucins can also be absorbed onto surfaces such as CS, and CS can be absorbed by mucins, forming an attached multilayer that is resistant to acids [102,103]. Meanwhile, CS can inhibit free radicals, which can damage the enamel structure [15,104]. The results of an enamel hardness test showed that nano-CS was better at maintaining the microhardness of the enamel surface than micro-chitosan and casein phosphopeptide-ACP (CPP-ACP) in an acidic environment. Furthermore, nano-CS reduced the porosity of enamel caused by acid, and the porosity was less than that of the micro-CS and CPP-ACP groups [15,104].

**Figure 5:**
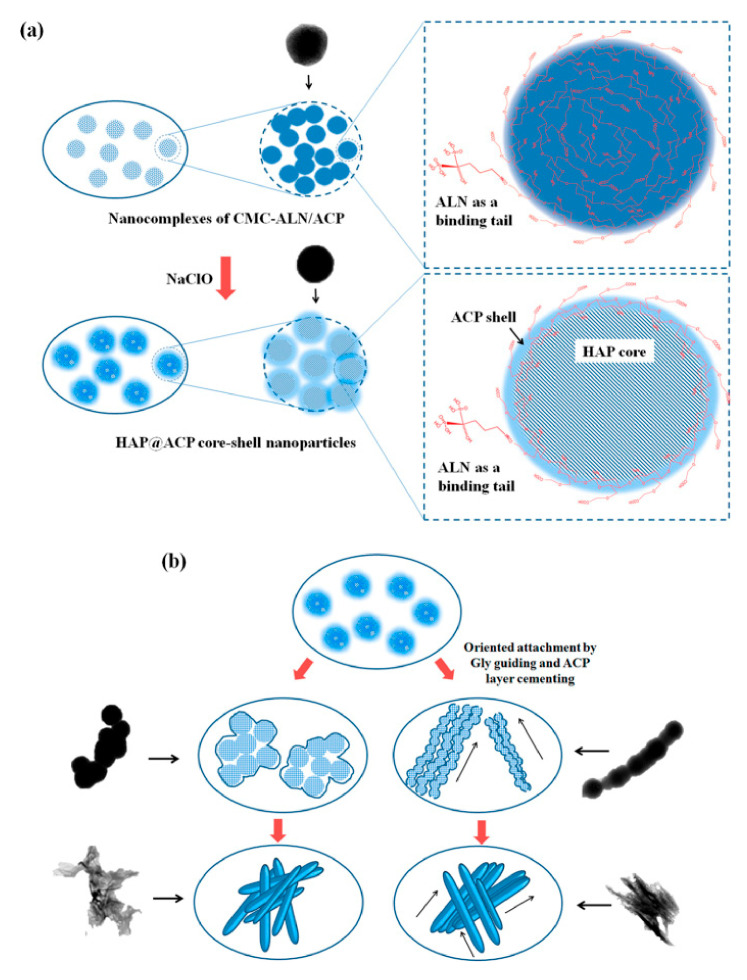
A schematic model of the formation of the nanocomplexes of carboxymethyl chitosan (CMC) conjugated with ALN. (**a**) Nalco hydrolyzes CMC to promote the conversion of ACP to HAP to form HAP@ACP core-shell nanoparticles; (**b**) Glycine-guided HAP@ACP core-shell nanoparticles’ linear aggregation. Reprinted with permission from ref. [98]. Copyright 2021 Springer Nature.

To improve the affinity of CS to the enamel surface, CS can be linked with alendronate (ALN), and the two phosphate groups of ALN can replace the two phosphate groups on the surface of HAP [98]. The residue of CS makes it difficult for newly formed ACP to transform into HAP; thus, the mechanical strength of remineralized enamel is lower than that of natural enamel [90,91,105]. Sodium hypochlorite and lysozyme can act on the 1,4-β-glycosidic bond of CS to form nanoparticles, eliminating the role of CS in stabilizing ACP. Nano-ACP formed by the cleavage of CS was rapidly transformed into a more stable HAP (Figure 5) [90,106]. Glycine guided the oriented growth of nanocomplexes to remineralize HAP, similar to natural enamel [98]. However, it remains unknown whether the mechanical properties of the mineral are affected by the cleavage of CS after hydrolysis by sodium hypochlorite and lysozyme. Lysozyme outside the system (such as lysozyme in saliva) may also disturb the electrostatic interaction between CS and proteins [107]. Meanwhile, the use of nano-CS could cause a slight browning of tooth color [108].

### 4.2. Dentin Remineralization

Dentin is the hard tissue foundation of human teeth. Approximately 70% of dentin is composed of plate-shaped irregular HAP nanocrystals with a small number of inorganic ions, such as Na^+^, Mg^2+^, and Zn^2+^, and regeneration ability [109]. In dentin, HAP can exist outside or inside collagen (COL) fibers [110]. Functional remineralization refers to the reintroduction of minerals into COL fibers and the partial or complete restoration of dentin properties [110]. The inorganic component of dentin is related to stiffness, while the organic component is responsible for toughness [111]. Therefore, the process of remineralization can increase the mechanical properties of dentin, including the hardness and modulus of elasticity [111]. There are positively and negatively charged amino acid clusters in the gap zone of the COL, forming a three-dimensional environment that can coordinate Ca^2+^ and PO_4_^3-^, to provide nucleation sites. Non-collagen proteins (NCPs), such as dentin matrix protein 1 and cementum protein 1, can self-assemble and induce the oriented mineralization of ACP at the nucleation site of COL, making the c-axis of HAP consistent with the c-axis of collagen fibers [112,113,114]. Intrafibrillar mineralization caused by NCPs is essential to increase the mechanical properties of dentin and prevent COL from inhibiting hydrolytic degradation [111,115].

Chitosan can form and stabilize ACP, and help ACP penetrate the gap zone of the COL to form a metastable crystalline phase, which finally forms a single apatite crystal. As a result, CS can be used as an analogue of NCP to promote the intrafibrillar remineralization of dentin [87,111]. The inclusion of CS and strontium-doped nano-HAP (Sr-nHAP) in remineralization paste significantly enhanced the antibacterial efficacy of the paste, and the paste had the same antibacterial effect as calcium hydroxide [87]. Meanwhile, the addition of CS to Sr-nHAP significantly improved the remineralization potential and mechanical properties of the demineralization dentin of Sr-nHAP [111].

## 5. Application of Nano-CS in Endodontics

**Figure 6:**
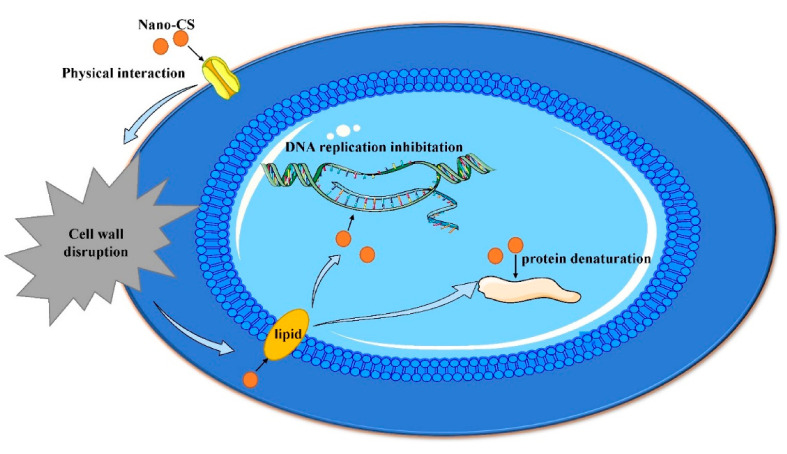
A schematic model of the antibacterial mechanism of nano-CS (Picture material cited from https://smart.servier.com/ (accessed on 10 September 2021).

The oral cavity is rich in bacteria, and interactions among bacteria affect their survival, function, and structure in the biofilm [8]. Imbalances in the microbiome may lead to plaque accumulation and bacterial biofilm growth, eventually causing dental caries, pulpitis, and apical periodontitis [116]. Therefore, it is important to inhibit oral pathogens and maintain the balance of oral microorganisms. Thus, CS has broad-spectrum antibacterial properties and a high killing rate, nano-CS has a higher penetration rate due to its smaller size, and nano-CS adheres to microbes in a shorter time due to its larger surface area [93,117,118]. Compared to CS, the inhibition zone of nano-CS for cariogenic bacteria in vitro was significantly higher (*p* < 0.05) [10]. Moreover, nano-CS can inhibit dual-species biofilms composed of *S. mutans* and *Candida albicans*, with an average diameter of 20−30 nm [8]. Cationic nano-CS diffuses through the cell wall of bacteria and is adsorbed on anionic particles (such as lipids) on the cell surface, cracking the cell membrane and eventually leading to bacteria death (Figure 6) [8,93,117]. In addition, nano-CS has sustained antibacterial properties, and the concentration of nano-CS has an inverse relationship with biofilm formation [9].

To reduce enamel demineralization during orthodontic treatment, nano-CS is added to orthodontic resin composites to inhibit *S. mutans*, *S. sanguis*, and *Lactobacillus acidophilus* [116]. The diameter of inhibition by diffusion was 5−8 mm for the three organisms.

The effective inhibition and killing of pathogenic bacteria in the root canal is key to the success of root canal therapy. Nano-CS can effectively inhibit the adhesion of *Enterococcus faecalis* to the root canal wall and eliminate this bacteria, which is common in apical periodontitis [119]. CS-coated poly(lactic co-glycolic) acid (PLGA) nanoparticles loaded with ciprofloxacin also inhibited *E. faecalis* and the formation of biofilm [6]. Therefore, nano-CS is a potential substitute for sodium hypochlorite in disinfection during root canal therapy [120].

However, it is challenging to completely eliminate the bacteria in the root canal system owing to the root canal’s complex anatomical structure and because bacteria enter dentinal tubules at varying depths (200–1500 μm) [121]. Hence, a medication with high permeability is essential for root canal treatment. Nanoparticles can be effectively delivered into the root canal system because of their size. Despite their antimicrobial properties, CS nanoparticles tend to agglomerate and congeal [122]. Therefore, nano-CSs should be transported into the root canal before this happens [120]. Conversely, nano-CS could crosslink with dentin COL and exhibit affinity with collagenase enzyme. Therefore, nano-CS has the ability to improve dentin stability in the long term [123]. As a result, nano-CS is a proven root canal irrigant.

## 6. Application of Nano-CS in Improving the Mechanical Properties of Dental-Restorative Materials

Owing to its small size, nano-CS can increase the surface area and charge density to form a physical and chemical combination between massive molecules and the CS effective chain. The formation of ionic bonds between CS itself and between CS and other polymers creates a continuous three-dimensional structure that increases the mechanical strength of polymers and can withstand a certain degree of force and pressure [61,124].

Meanwhile, nano-CS can be used to fill the gap between the particles of dental materials and the gap between dental materials and dental tissue, to improve the physical and chemical properties of dental materials.

### 6.1. Glass Ionomer Cement (GIC)

GIC is a commonly used therapeutic material in stomatology [125,126]. Conventional GIC can chemically adhere to hard tooth structures, release fluoride, resist bacteria and caries, and induce remineralization [125]. High-molecular nano-CS was added to the interface of resin-modified GIC and GIC and then the materials were applied to the premolar with a Class I cavity. The addition of nano-CS improved the adhesion of GIC to dentin [127]. The most likely reason for this is that the nano-CS reduced the interfacial tension between the different components of the GIC and increased the surface and charge density, leading to better interactions between the nano-CS and the components of the GIC [126,128]. Because of its smaller size, nano-CS can fill the small voids between larger GIC component particles and form more physical and chemical bonds with polyacrylic acid polymers [126]. Therefore, 10 wt% nano-CS-modified GIC had significantly higher compressive strength values, flexural strength, wear resistance (due to a better integrated interface between the glass particle and polymer matrix bonding). and fluoride release, compared to conventional GIC [129]. It can be inferred that adding nano-CS to GIC can improve its mechanical properties and anti-caries properties.

### 6.2. Dental Sealants

The instability of CS-free amino groups under acidic conditions limits the application of CS in many fields. The N-acylation of CS could decrease the instability. Owing to poor water solubility, fully N-acylated CS nanoparticles could not be obtained by ionotropic gelation [130]. N-acylated CS was stable over a wide pH range, which confirmed it as a nano-reinforcement material for composites [130]. In this case, ultrafine milling was an effective method for obtaining nanoparticles of N-acylated chitosan. The modified CS powder was ultrafine-milled with zirconia balls (1–5 mm). The nanopowder obtained from the ultrafine milling was extracted in acetone, and ultrasonication was introduced to reduce the particle size. The solvent was then removed by a high vacuum force [130,131]. By prolonging the milling time, a finer particle size can be obtained. At the same time, this procedure ensures that the nano-CS does not contain residual solvents that will cause toxicity [131]. Pit and fissure sealants with nanoparticles of hydrophobic N-(2-ethylhexanoyl) CS, with an average diameter of 320–670 nm, showed slight improvement in wear behavior compared to an unmodified group [131]. Nanoparticles, which replace micro-sized fillers, ensured that sealants completely penetrated the microspaces of enamel to improve its mechanical properties [132,133].

### 6.3. Base Resin

To improve the poor mechanical properties of heat-polymerized polymethylmethacrylate (PMMA), a popular denture base material, researchers often add additivest [134]. The addition of nano-CS increased transverse strength, impact strength, and color stability [61,135,136]. The transverse strength increased the tension of the denture base, resulting in cracks in the denture base [137]. Because of its impact strength, fractures of the acrylic resin denture base after several years of usage were 68% [138]. However, 1% nano-CS was the ideal concentration of transverse strength, impact strength, and color stability for high-viscosity CS solutions with different densities [135,136]. In the modified materials containing 1.5% nano-CS, it was difficult for the nano-CS to diffuse into small voids in PMMA, resulting in relatively few physical and chemical bonds, eventually causing decreased transverse strength [61,135]. Nano-CS decreases porosity, affecting the density and strength of the material [61]. Meanwhile, the membranous porosity of CS decreases with increasing concentrations of CS [61].

## 7. Prospective Applications of Chitosan-Based Nanomaterials in Dentistry

Chitosan is the only cationic alkaline polysaccharide polymer in nature and has been widely used in biomedical engineering. Chitosan-based nanomaterials have many advantages as drug carriers for soft tissue diseases, bone tissue engineering, and dental hard tissue remineralization. These include high surface area and charge density, biocompatibility, non-toxicity, and degradability. In particular, the amino or hydroxyl groups on the chitosan nanoparticles were easily modified to achieve precise drug release. Furthermore, chitosan nanoparticles improve the adhesion and mechanical properties of dental restorative materials by interacting with various molecules through physical blending (hydrogen bonds) or chemical combination. In addition, using the antibacterial properties of nano-CS, chitosan nanoparticles have great potential for application in oral healthcare products.

Significant progress has been made in the research into nano-CS in stomatology, but much research is required to determine its clinical application. There are numerous problems, including the following: 1. Because the gel characteristics of chitosan increase as the scale becomes smaller, nano-CS does not appear in the dentin tubule. which limits the application of nano-CS in dentin mineralization, closed dentin tubules, and root canal treatment. 2. The mechanical strength of CS after gelation is low; therefore, it is not suitable for repairing bone tissue defects alone. 3. Most of the surfactants used in the preparation of nano-CSs are highly toxic, and most nano-CSs are prepared in acidic liquids. Moreover, toxic and acidic environments are not suitable osteogenic microenvironments. Currently, efficient methods are being designed to find new non-toxic reagents to prepare stable nano-CS systems in dentistry, and then to obtain nano-CSs with distinguished dispersion to verify their cytotoxicity and therapeutic effects. This will allow a breakthrough in the application of nano-CSs in stomatology.

## 8. Conclusions

Combined with nanotechnology, chitosan shows an outstanding performance in stomatology application, but it is still in the preliminary exploration stage for the majority of oral therapy research. Furthermore, although most of studies showed that nano-CS offers excellent application potential, many barriers remain to its popularization in clinical practice and its ability to achieve appropriate therapeutic effects. Future research should focus on the stability, non-toxicity, and biodegradability of nano-CSs. We believe that with the development of relevant technologies in biomaterial science and molecular biology, chitosan and its nanoparticles will be widely used in stomatology.

## Data Availability

Not applicable.

## Abbreviations

Chitosan (CS), bacterial cellulose (BC), hydroxyapatite (HAP), glass ionomer cement (GIC), polymerized polymethylmethacrylate (PMMA), calcium phosphate cements (CPCs), glycerol phosphate (GP), risedronate (RIS), mesenchymal stem cells (MSCs), alkaline phosphatase (ALP), silk fibroin (SF), Poly(ɛ-caprolactone)-poly(ethylene glycol)-poly(ε-caprolactone) (PCEC), human dental pulp stem cells (hDPSCs), modified simulated body fluid (m-SBF), electrophoretic deposition (EPD), pulse electrodeposition (PED), electrospinning (ESP), amelogenin (AMEL), alendronate (ALN), collagen (COL), noncollagen proteins (NCPs), amorphous calcium phosphate (ACP), recombinant Human Keratinocyte Growth Factor (rHuKGF), hydroxy modified glucose chitosan (HGC), trichloroacetic acid (TCA), epidermal growth factor (EGF), Integrin 1 (ITGB1)
